# A *Sclerotinia sclerotiorum* Transcription Factor Involved in Sclerotial Development and Virulence on Pea

**DOI:** 10.1128/mSphere.00615-18

**Published:** 2019-01-23

**Authors:** Hyunkyu Sang, Hao-Xun Chang, Martin I. Chilvers

**Affiliations:** a Department of Plant, Soil and Microbial Sciences, Michigan State University, East Lansing, Michigan, USA; Carnegie Mellon University

**Keywords:** *Sclerotinia sclerotiorum*, gene silencing, pea, sclerotial development, transcription factor, virulence

## Abstract

Sclerotinia sclerotiorum is a plant-pathogenic ascomycete fungus and infects over 400 host plants, including pea (Pisum sativum L.). The fungus causes white mold on pea, and substantial yield loss is attributed to the disease. To improve white mold management, further understanding of S. sclerotiorum pathogenicity is crucial. In this study, 389 transcription factors (TFs) were mined from the complete genome sequence of S. sclerotiorum and their *in planta* expression patterns were determined in susceptible and partially resistant pea lines and compared to *in vitro* expression patterns on culture medium. One of the transcription factors was significantly induced *in planta* at 24 and 48 h postinfection compared to the expression *in vitro*. This putative C6 transcription factor of S. sclerotiorum (SsC_6_TF1) was knocked down using a gene-silencing approach to investigate its functions in vegetative growth and sclerotial development as well as its virulence and pathogenicity in pea. While the SsC_6_TF1 knockdown mutants had hyphal growth rates identical to those of the wild-type strain and were capable of infection, the knockdown mutants produced no sclerotia or significantly fewer and smaller sclerotia on the culture medium and exhibited reduced virulence on both pea lines. This study profiled genome-wide expression for S. sclerotiorum transcription factors *in planta* and *in vitro* and functionally characterized a novel transcription factor, SsC_6_TF1, which positively regulates sclerotial development and virulence on pea. The finding provides molecular insights into S. sclerotiorum biology and interaction with pea and other economically important crops.

**IMPORTANCE** White mold, caused by Sclerotinia sclerotiorum, is a destructive disease on important legume species such as soybean, dry bean, and pea. This study investigated expression levels of transcription factors in S. sclerotiorum *in planta* (pea lines) and *in vitro* (culture medium). One transcription factor displaying high expression *in planta* was found to be involved in sclerotial development and virulence on pea. This report provides a new understanding regarding transcription factors of S. sclerotiorum in development and virulence.

## Abstract (precis)

White mold, caused by Sclerotinia sclerotiorum, is a destructive disease on important legume species such as soybean, dry bean, and pea. This study investigated expression levels of transcription factors in S. sclerotiorum *in planta* (pea lines) and *in vitro* (culture medium). One transcription factor displaying high expression *in planta* was found to be involved in sclerotial development and virulence on pea. This report provides a new understanding regarding transcription factors of S. sclerotiorum in development and virulence.

## OBSERVATION

The plant-pathogenic fungus Sclerotinia sclerotiorum, the causal agent of white mold, has a wide range of hosts (1) and causes significant yield loss on crops such as soybean, dry bean, and pea (2–4). In the Pacific Northwest and other parts of the United States, irrigated and dry land peas are often seriously damaged by white mold (5, 6). Since complete resistance to S. sclerotiorum is not present in pea (7), studies in pea-S. sclerotiorum interaction are needed to advance white mold management through understanding mechanisms of plant partial resistance and virulence factors as well as pathogenicity factors of S. sclerotiorum. Transcriptomic analyses using RNA-seq have become a powerful approach to explore plant resistance to white mold (8, 9) and virulence mechanisms of S. sclerotiorum (10, 11). However, most studies exploring *Sclerotinia* virulence and pathogenicity have focused on oxalic acid, effectors, and cell-wall degrading enzymes and few studies have focused on fungal transcription factors (TFs) involved in virulence (12).

**FIG 1:**
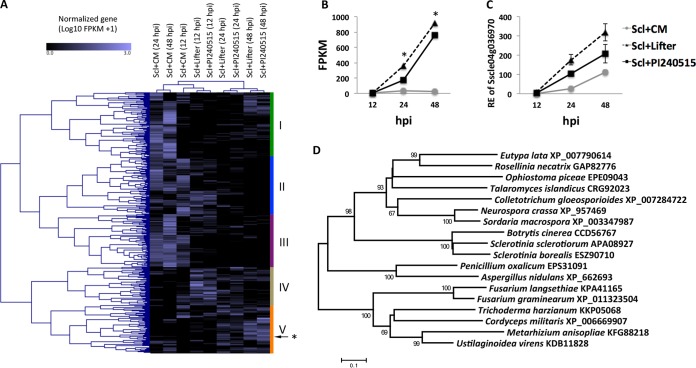
Expression patterns of Sclerotinia sclerotiorum transcription factors and a candidate S. sclerotiorum virulence factor, SsC_6_TF1. (A) A clustered heat map showing *in planta* and *in vitro* expression of 389 putative transcription factors in the pea lines “Lifter” and PI240515 and in the culture medium (CM) at 12, 24, and 48 hpi. An asterisk (*) indicates a candidate virulence factor SsC_6_TF1 (Sscle04g036970) in group V, which has a tendency toward higher expression *in planta* than *in vitro*. (B) Expression levels of SsC_6_TF1 *in planta* in RNA-Seq were highly induced at 24 and 48 hpi but not *in vitro* (*, FDR-adjusted *P* < 0.05). (C) Expression patterns of SsC_6_TF1 *in planta* and *in vitro* were confirmed by qPCR analysis. RE, relative expression. (D) Neighbor-joining tree for amino acid sequences of SsC_6_TF1 (Sscle04g036970; APA09027) and its orthologs in other fungi. The scale bar represents the number of amino acid substitutions per site. Bootstrap values are based on 1,000 iterations.

The current study investigated the genome-wide TFs in S. sclerotiorum and their *in planta* expression patterns during infection of a susceptible pea cultivar (“Lifter”) and a partially resistant pea line (PI240515) in contrast to *in vitro* expression on culture medium (CM; potato dextrose agar [PDA]). In this study, a total of 389 putative TFs were identified from the complete and gapless S. sclerotiorum genome (13). Transcriptomes of these TFs were analyzed using the standard RNA-Seq pipeline Tuxedo Suite, and the TFs were clustered into five groups corresponding to expression levels (Fig. 1A). Group I consisted of 99 TFs that were most induced *in vitro* at 24 and 48 h postinoculation (hpi). Among the group I TFs, *in vitro* expression levels of C2H2 type zinc finger TF (sscle_01g002350) at 24 and 48 hpi were significantly higher than the expression levels at 12 hpi. The *in vitro* expression levels of TF (sscle_01g002350) at 24 and 48 hpi were also higher than *in planta* expression levels at 24 and 48 hpi. Eighty-three TFs in group II were most highly expressed *in vitro* at 12 hpi. Group III contains 79 TFs, and most of those TFs were induced *in vitro* at all time points. Two TFs (sscle_02g017040 and sscle_01g004470) in this group were more highly expressed *in vitro* than *in planta* at all time points. These two TFs are annotated with a C2H2 zinc finger domain and a basic helix-loop-helix dimerization domain, respectively. Group IV, containing 57 TFs, showed the highest expression *in planta* at 12 hpi; among them, the *in planta* expression levels of C2H2 type TF (sscle_06g049860) at 12 hpi were significantly higher than the *in vitro* and *in planta* expression levels at 24 hpi. The *in planta* expression levels of group IV TFs generally decreased over time, indicating their potential functions in establishing early infection. On the other hand, group V, containing 71 TFs, showed *in planta* expression levels that gradually increased from 12 to 48 hpi (Fig. 1A). Among the TFs, one putative S. sclerotiorum C6 TF (SsC_6_TF1; Sscle04g036970; SS1G_002671) showed higher expression in the susceptible pea variety Lifter than in the resistant variety PI240515 and showed significantly higher expression *in planta* than *in vitro* at 24 and 48 hpi (Fig. 1B). Therefore, it appeared to be the best candidate for further study of regulation of S. sclerotiorum development and virulence on pea. Expression patterns of two representative TFs (sscle_01g002350 and Sscle04g036970) were confirmed by quantitative PCR (qPCR) (Fig. 1C; see also Fig. S1 in the supplemental material). SsC_6_TF1 shares 75% and 70% amino acid identity with TFs from *Sclerotinia borealis* (GenBank accession no. ESZ90710) and *Botrytis cinerea* (GenBank accession no. CCD56767), respectively. Phylogenetic analysis revealed that orthologous TFs in genomes of ascomycete fungi remained evolutionary conserved (Fig. 1D).

10.1128/mSphere.00615-18.1 FIG S1 Expression of Sclerotinia sclerotiorum transcription factor (sscle_01g002350) in the pea lines “Lifter” and PI240515 and in the culture medium (CM) at 12, 24, and 48 hpi generated from (A) RNA-Seq analysis and (B) qPCR analysis. Download FIG S1, PDF file, 0.09 MB. Copyright © 2019 Sang et al. 2019 Sang et al. This content is distributed under the terms of the Creative Commons Attribution 4.0 International license.

**FIG 2:**
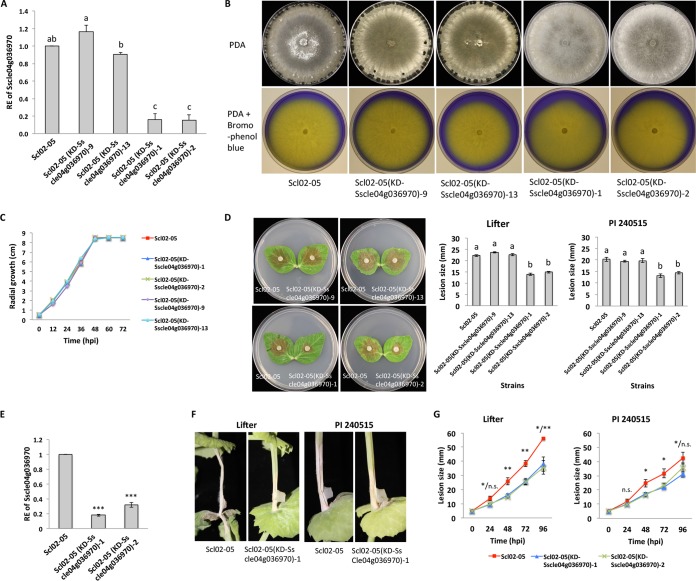
SsC_6_TF1 (Sscle04g036970) regulates sclerotial development and virulence on pea. (A) Relative expression (RE) levels of Sscle04g036970 in wild-type strain Scl02-05, two control mutants [Scl02-05(KD-Sscle04g036970)-9 and Scl02-05(KD-Sscle04g036970)-13], and two SsC_6_TF1 knockdown mutants [Scl02-05(KD-Sscle04g036970)-1 and Scl02-05(KD-Sscle04g036970)-2] grown in PDB for 48 hpi. The mean values followed by different letters on the same graph are significantly different according to Fisher’s least-significant-difference test at *P* = 0.05. (B) The wild-type strain and four mutants were grown on PDA for 6 days and on PDA amended with bromophenol blue (50 mg liter^−1^) for 2 days. (C) Radial growth of the wild-type strain and four mutants on PDA. (D) Pathogenicity assays of wild-type strain and four mutants on the detached leaves of Lifter. The photos were taken at 22 hpi. Lesion sizes caused by the wild-type strain and four mutants on the detached leaves of pea lines Lifter and PI240515. Mean values followed by different letters on the same graph are significantly different according to Fisher’s least-significant-difference test at *P* = 0.05. (E) Relative expression of Sscle04g036970 in the Scl02-05 wild-type strain and two SsC_6_TF1 knockdown mutants [Scl02-05(KD-Sscle04g036970)-1 and Scl02-05(KD-Sscle04g036970)-2] on Lifter at 48 hpi. (***, *P* < 0.001). (F) Pathogenicity assays of the wild-type strain and SsC_6_TF1 knockdown mutant on the stems of two pea lines. The photos were taken at 48 hpi. (G) Lesion sizes caused by the wild-type strain and two SsC_6_TF1 knockdown mutants on the stems of pea lines Lifter and PI240515 [Scl02-05(KD-Sscle04g036970)-1/Scl02-05(KD-Sscle04g036970)-2] (n.s., not significant; *, *P* < 0.05; **, *P* < 0.01).

SsC_6_TF1 knockdown mutants were generated from wild-type strain Scl02-05 using a gene-silencing approach. Among 15 transformants selected from regenerate medium amended with hygromycin (100 μg ml^−1^), five transformants showed a reduced number of sclerotia. Four mutants displaying different levels of sclerotial production were chosen for further experiments. The expression of SsC_6_TF1 (Sscle04g036970) was confirmed to be significantly downregulated in two SsC_6_TF1 knockdown mutants [Scl02-05(KD-Sscle04g036970)-1 and Scl02-05(KD-Sscle04g036970)-2] compared to the other two mutants [Scl02-05(KD-Sscle04g036970)-9 and Scl02-05(KD-Sscle04g036970)-13] and the wild-type strain (Fig. 2A). Four mutants and the wild-type strain were grown on PDA for 6 days to examine hyphal growth and sclerotial formation and on PDA amended with bromophenol blue for 2 days to assay oxalic acid production. Interestingly, while the growth rates and oxalic acid production levels of the four mutants and the wild-type strain were not different (Fig. 2B and C), two SsC_6_TF1 knockdown mutants [Scl02-05(KD-Sscle04g036970)-1 and Scl02-05(KD-Sscle04g036970)-2] produced no sclerotia or significantly fewer sclerotia on PDA, even after 1 month, than the other two mutants and wild-type strain (*P* < 0.0001) (Fig. 2B). The pathogenicity of the two SsC_6_TF1 knockdown mutants was tested on pea using a detached-leaf pathogenicity/virulence assay. The two SsC_6_TF1 knockdown mutants showed reduced virulence on detached leaves of both pea lines compared to the other two mutants and wild-type strain (*P* < 0.0001) (Fig. 2D). Also, the expression levels of SsC_6_TF1 (Sscle04g036970) in the SsC_6_TF1 knockdown mutants and the wild-type strain were quantified *in planta* on susceptible pea line Lifter. The expression of Sscle04g036970 was confirmed to be significantly downregulated in SsC_6_TF1 knockdown mutants [Scl02-05(KD-Sscle04g036970)-1 and Scl02-05(KD-Sscle04g036970)-2] compared to the wild-type strain (*P* < 0.0001) (Fig. 2F). Both SsC_6_TF1 knockdown mutants caused significantly smaller lesions than the wild-type strain on stems of both pea lines (Fig. 2F and G). Accordingly, SsC_6_TF1 is not required for vegetative hyphal growth and pathogenicity, but it is a positive regulator for sclerotial development and virulence on pea.

TFs are essential to regulate gene expression in living organisms. Amselem et al. (14) reported 330 TFs in S. sclerotiorum, and, in this study, an additional 59 putative TFs were mined from the complete genome sequence (13). Because S. sclerotiorum initiates sclerotial development when its hyphae touch the edge of culture plates, group I TFs with late expression may be involved in sclerotial development. High *in vitro* expression levels of two TFs in group III indicated their potential function in vegetative growth and/or sclerotial development. In this study, a novel C6 TF of S. sclerotiorum (SsC_6_TF1) was found to positively regulate sclerotial development. As several signal transduction pathways (e.g., cAMP-dependent and pH-dependent signaling pathways) are involved in sclerotial formation (12, 15), further investigation on the roles of SsC_6_TF1, such as its potential interaction with other sclerotial-development-related genes (e.g., the gene encoding Ssp1) (16), is needed to decipher mechanisms of sclerotial formation.

Kabbage et al. (17) suggested that S. sclerotiorum has a biotrophic lifestyle in early infection stages and switches to a necrotrophic lifestyle once the fungus acquires nutrients from tissue of dead plants. Expression of (group IV) TFs in the early infection stage might be associated with the biotrophic stage. In addition, expression of group V TFs, including SsC_6_TF1, at 24 and 48 hpi might regulate fungal virulence in the necrotrophic stage. The finding of reduced virulence of SsC_6_TF1 knockdown mutants supports the idea of the role of SsC_6_TF1 in *Sclerotinia* virulence on pea, but additional understanding of SsC_6_TF1 regulatory mechanisms in the necrotrophic stage is needed. Moreover, SsC_6_TF1 was more highly expressed in the susceptible pea cultivar than in the partially resistant pea line at 24 and 48 hpi, but when SsC_6_TF1was knocked down, the mutants caused lesions of similar sizes on both pea lines. Identifying regulons of SsC_6_TF1 and studying interaction of the TF’s downstream genes with pea resistance genes will provide molecular insights into sclerotial development, virulence, and plant resistance that may apply not only to pea but also to other economically important crops. Although SsC_6_TF1 was identified as a C_6_ transcription factor on the basis of a BLAST search, no conserved domains were detected using a conserved domain search. Further investigation of this putative TF is needed to characterize its mechanistic functions such as the localization of the TF and binding of the TF to promoter regions of target genes.

### Pea (Pisum sativum L.) lines and Sclerotinia sclerotiorum strains.

Two pea lines, a partially resistant line (PI240515) and a susceptible line (Lifter), were used as host plants (8). S. sclerotiorum strain Scl02-05 (8) isolated from pea was used as the wild-type strain.

### RNA-Seq analysis.

For *in planta* samples, pea lines PI240515 and Lifter at the fourth-node leaf axil were inoculated with plugs from the edge of an S. sclerotiorum strain (Scl02-05) colony on PDA. For *in vitro* samples, the plugs from the margin of the Scl02-05 colony were inoculated on fresh PDA plates and samples for RNA extraction were taken from the growing colony margin at each time point. The symptoms were not observed in the two pea lines at 12 hpi, but the lesion was observed on both pea lines at 24 hpi. The susceptible line Lifter exhibited a larger stem lesion than the resistant line PI240515, and the Lifter plants showed lodging but not PI240515. At 48 hpi, the lesions observed on the stem and leaf were larger on Lifter than on PI240515; additional phenotype details can be found in a report by Chang et al. (8). Two biologically replicated *in planta* and *in vitro* samples were harvested at 12, 24, and 48 hpi, and RNA was extracted and sequenced as described previously by Chang et al. (8). The raw RNA sequences were deposited in NCBI SRA database. The RNA-Seq analysis was conducted using a standard pipeline (Tuxedo Suite), including TopHat2 and Cufflinks modules (18). The complete genome of S. sclerotiorum (BioProject no. PRJNA348385) (13) was used as a reference genome. TFs were mined from the complete genome sequences of S. sclerotiorum using BLAST2GO with a query list from the previously identified TFs by Amselem et al. (14). The heat map was generated using Genesis software (version 1.7.7) with a complete linkage clustering method (http://genome.tugraz.at) (19), and the normalized gene expression [log_10_(FPKM + 1), where FPKM is fragments per kilobase per million] of 389 putative TFs of S. sclerotiorum.

### RNA extraction and qPCR analysis.

Three biologically replicated *in planta* and *in vitro* samples were prepared in this study by the method described above to validate expression patterns of TFs of S. sclerotiorum from RNA-Seq by quantitative PCR (qPCR). Briefly, total RNA of harvested samples was extracted using a Zymo RNA extraction kit (Zymo Research, Irvine, CA). cDNA synthesis from total RNA was conducted using a QuantiTect reverse transcription kit (Qiagen, Germantown, MD), and the expression of target genes was quantified by qPCR using SYBR green PCR Master Mix (Thermo Fisher Scientific). The actin gene (*SSactin*; SS1G_08733) was selected as a housekeeping gene. Primers used in qPCR are described in Table S1 in the supplemental material. The comparative threshold cycle (*C_T_*) method was used for calculating relative gene expression levels (20).

10.1128/mSphere.00615-18.2 TABLE S1 Primers used in this study. Download Table S1, PDF file, 0.4 MB. Copyright © 2019 Sang et al. 2019 Sang et al. This content is distributed under the terms of the Creative Commons Attribution 4.0 International license.

### Plasmid construction and generation of SsC_6_TF1 knockdown mutants.

Plasmid pYHN3-ptrpC-dsRNAXDR1 (21) was used for SsC_6_TF1 knockdown. Each 200-bp fragment of sense and antisense in SsC_6_TF1(Sscle04g036970) amplified from cDNA of wild-type strain was inserted into pYHN3-ptrpC-dsRNAXDR1 to generate the plasmid pYHN3-ptrpC-dsRNATF. The inserted fragments were confirmed by Sanger sequencing in the Research Technology Support Facility of Michigan State University (East Lansing, MI). The generated plasmid DNA (5 μg) was transformed into protoplasts of wild-type strain. Protoplast generation and polyethylene glycol (PEG)-mediated transformation were conducted according to a method previously described by Sang et al. (22). The primers used for plasmid construction and sequencing are described in Table S1.

### Validation and characterization of SsC_6_TF1 knockdown mutants.

Among 15 hygromycin-resistant transformants, four transformants [Scl02-05(KD-Sscle04g036970)-1, Scl02-05(KD-Sscle04g036970)-2, Scl02-05(KD-Sscle04g036970)-9, and Scl02-05(KD-Sscle04g036970)-13] with different levels of sclerotial production were selected for the validation of SsC_6_TF1 knockdown. Mycelia of four transformants and the wild-type strain grown on potato dextrose broth (PDB) for 48 h were harvested for RNA extraction. Also, two SsC_6_TF1 knockdown mutants [Scl02-05(KD-Sscle04g036970)-1 and Scl02-05(KD-Sscle04g036970)-2] and the wild-type strain were inoculated on Lifter at the third-node leaf axil. After 48 h of incubation, the infected lesion was harvested. RNA extraction and qPCR analysis of the samples were performed for the validation of SsC_6_TF1 knockdown mutants. To examine growth rates, sclerotial formation, and oxalic acid production, the four mutants and the wild-type strain were grown on PDA and on PDA amended with bromophenol blue (50 mg liters^−1^) at 25°C in the dark. Colony diameters on PDA were measured in two perpendicular directions at 12, 24, 36, and 48 hpi using Digimatic calipers (Mitutoyo, Japan). Photos of *Sclerotinia*-colonized PDA plates were taken after 6 days of incubation to document sclerotial formation (or lack thereof). *Sclerotinia*-colonized PDA amended with bromophenol blue were photographed after 2 days of incubation to document oxalic acid production. The experiment was performed twice with three replicate plates per experiment.

### Plant growth and pathogenicity tests of SsC_6_TF1 knockdown mutants.

Pea seeds of PI240515 and Lifter were sown into a perlite soil mix (Suremix Perlite; Michigan Grower Products Inc., MI) in pots (6.5 by 6.5 by 8.8 cm) and grown for 18 days in a growth chamber at 25°C with a 14-h light period and were watered as necessary. PDA plugs from the edge of 36-h-old colonies of the two SsC_6_TF1 knockdown mutants and the wild-type strain were inoculated on detached leaves of PI240515 or Lifter. PDA plugs were also inoculated onto PI240515 or Lifter seedlings at the third-node leaf axil. Photos of detached S. sclerotiorum*-*infected leaves were taken, and the lesion diameter was measured in two perpendicular directions per leaf at 22 hpi. S. sclerotiorum*-*infected pea stems were photographed at 48 hpi, and the lesions were measured at 24, 48, 72, and 96 hpi. The experiment was conducted twice with three replicate detached leaves or pots per experiment.

### Statistical analysis.

In the RNA-Seq data, TFs showing differentially expression [greater than a 1.5-fold (log_2_) difference at a false-discovery rate (FDR) of 5%] under the different sample conditions were identified using cuffdiff (http://cole-trapnell-lab.github.io/cufflinks/cuffdiff/, v2.2.1). To test the differences in the numbers of sclerotia, the expression levels of Sscle04g036970, or the sizes of the stem lesions of the wild-type strain and each mutant, analysis of variance (ANOVA) was conducted. The separation of Sscle04g036970 expression values or sizes of leaf lesion from the four mutants and the wild-type strain was done using Tukey’s honestly significant difference (HSD) test (α = 0.05). The statistical analyses (ANOVA and Tukey’s HSD) were performed using JMP software, version 14.0 (SAS Institute Inc., NC).

### Data availability.

The raw RNA sequences were deposited in the NCBI SRA database, and the sequences are available under BioProject accession number PRJNA261444.
