# Supplementary material for: A Sclerotinia sclerotiorum Transcription Factor Involved in Sclerotial Development and Virulence on Pea
Source: mSphere. 2019 Jan 23;4(1):e00615-18. doi: 10.1128/mSphere.00615-18 (PMC6344603; doi:10.1128/mSphere.00615-18)
Supplement: FIG S1 [file mSphere.00615-18-sf001.pdf]

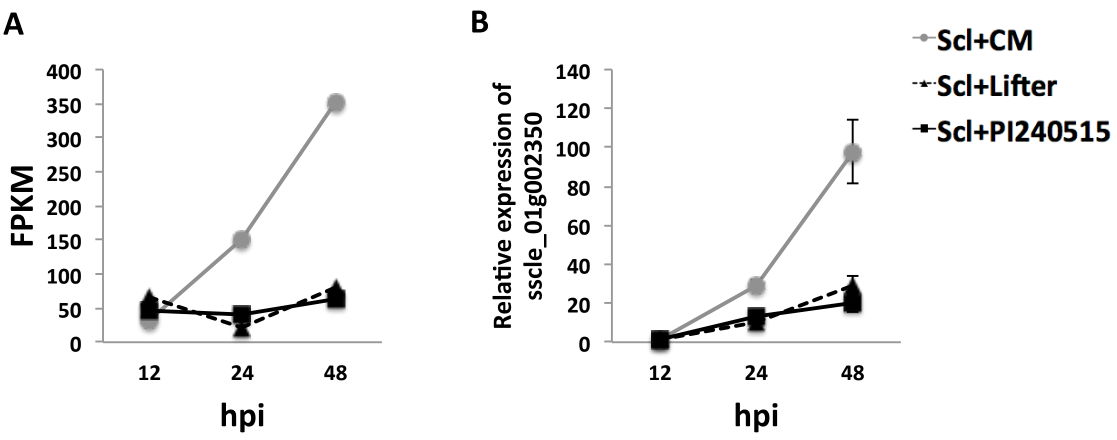

Fig. S1. Expression of *Sclerotinia sclerotiorum* transcription factor (sscle_01g002350) in the pea lines 'Lifter' and PI240515 and in the culture medium (CM) at 12, 24, and 48 hpi generated from (A) RNA-seq analysis and (B) qPCR analysis.
