# Supplementary material for: A Sclerotinia sclerotiorum Transcription Factor Involved in Sclerotial Development and Virulence on Pea
Source: mSphere. 2019 Jan 23;4(1):e00615-18. doi: 10.1128/mSphere.00615-18 (PMC6344603; doi:10.1128/mSphere.00615-18)
Supplement: TABLE S1 [file mSphere.00615-18-st001.pdf]

**Table S1.** Primers used in this study.

| Primers name | Primer sequence (5'-3') |
|---|---|
| F_AscI_04g036970 | ATTTGGCGCGCCACTATTGTTCTATTCTCTCT |
| R_SwaI_04g036970 | CGCGATTTAAATGTCTTGAACTTTCCGCATCT |
| F_BamI_04g036970 | ATTTGGATCCGTCTTGAACTTTCCGCATCT |
| F_BamI_04g036970 | ATGCGGATCCACTATTGTTCTATTCTCTCTCAGC |
| F_dsseq_check | CCATGGAAGCTTGGTACCGAGCTCTATTTTTACA |
| R_dsseq_check | GAGATCCTGAACACCATTTGTCTCAACTCCGG |
| QF_SSactin | CAACCCAAAGTCCAACAGAGA |
| QR_SSactin | GTACGACCGGAAGCGTAAAG |
| QF_04g036970 | CTTCCTTTGGCGAGCTATCA |
| QR_04g036970 | GTCGGGTAATCCTTGACTCTTC |
| QF_01g002350 | GCCCTTCAGAACCTTGTAGTT |
| QR_01g002350 | CCTTGCTTCTCTTCTTCCTCTC |
